# Validation of the international consultation on incontinence modular questionnaire – female lower urinary tract symptoms (ICIQ-FLUTS) into brazilian portuguese

**DOI:** 10.1590/S1677-5538.IBJU.2019.0234

**Published:** 2020-01-13

**Authors:** Priscylla Helouyse Angelo, Neila Alves de Queiroz, Alethéa Cury Rabelo Leitão, Gabriela Marini, Maria Thereza Micussi

**Affiliations:** 1 Departamento de Fisioterapia, Universidade Federal do Rio Grande do Norte (UFRN), Natal, Rio Grande do Norte, Brasil; 2 Centro de Ciências da Saúde, Universidade do Sagrado Coração (USC), Bauru, SP, Brasil

**Keywords:** Lower Urinary Tract Symptoms, Validation Studies [Publication Type], Women

## Abstract

**Purpose:**

To translate, adapt and validate the International Consultation on Incontinence Modular Questionnaire on Female Lower Urinary Tract Symptoms ICIQ-FLUTS for the Brazilian female population.

**Materials and Methods:**

A translation of the questionnaire into Brazilian Portuguese was made followed by an adaptation for better understanding by native speakers. After that, the ICIQ-FLUTS was answered by eighty volunteers (n=80) twice (for interviewers 1 and 2) with an interval of 30 minutes between them. Furthermore, after 15 days from the evaluation, the participants answered the ICIQ-FLUTS again in order to verify the questionnaire stability over time. The questionnaires Utian Quality Of Life (UQOL) and International Consultation on Incontinence Questionnaire - Short Form (ICIQ-SF), which are validated in Brazil were also applied to perform the validation.

**Results:**

The result of the Cronbach α coefficient of the instrument presented a value of 0.832. The values for test-retest were 0.907 (inter-observer) and 0.901 (intra-observer). The correlation between ICIQ-FLUTS (score I - domain of urinary incontinence) with the ICIQ-SF (final score) was strong and positive (r=0.836, p=0.000). In addition, the ICIQ-FLUTS showed moderate and negative correlation with the total score of UQOL (r=-0.691, p=0.017).

**Conclusion:**

The Portuguese version of the ICIQ-FLUTS questionnaire showed strong correlation to ICIQ-SF questionnaire and satisfactory values to test-retest and internal consistency.

## INTRODUCTION

Lower urinary tract symptoms (LUTS) are defined as changes in the storage, voiding and/or post-voiding phase of the urination process, for example, overactive bladder syndrome, nocturia, urinary incontinence (UI), among others (1). LUTS is a clinical condition with a high prevalence in the general population, exceeds 60% in Europe and North America (2, 3), similar results to those found in a Brazilian study (59% in women aged ≥40 years) (4).

The presence of the symptoms mentioned above can negatively affect the women's quality of life, therefore, LUTS is considered a serious and relevant health problem for women, and its evaluation is important for further treatment (4, 5).

The International Consultation on Incontinence Modular Questionnaire on Female Lower Urinary Tract Symptoms (ICIQ-FLUTS) is a tool which allows to evaluate and quantify LUTS and their impact on quality of life (6). The instrument is derived from the Bristol Female Lower Urinary Tract Symptoms Questionnaire originally developed in English. The final version removed issues in relation to sexuality and included a scoring system divided into three domains: filling, voiding and incontinence (7).

There are validated questionnaires in Brazil used to evaluate UI (8–10), however, the involuntary loss of urine is just one of several types of LUTS. The lack of instruments which evaluate LUTS may difficult its investigation, treatment, as well as the knowledge about the impact of the disease on the quality of life.

Many studies including randomized and controlled clinical trials apply validated questionnaires to evaluate the outcomes of researches. The ICIQ-FLUTS approaches a wide diversity of urinary symptoms presenting level of evidence “A”, thus, highly recommended by the International Consultation on Incontinence (ICI). Furthermore, it is brief and presents a good acceptation in clinical practice (11).

Due to the high prevalence of LUTS in women, it is evident that there is a need to create new tools for evaluating urinary symptoms in a broader way. The translation and validation for other languages provide further comparison between studies. In relation to this, the objective of this study was to translate, adapt and validate the ICIQ-FLUTS for the Brazilian female population.

## MATERIALS AND METHODS

A prospective validation study was realized in Brazil. This study aims the translation, adaptation and validation of the International Consultation on Incontinence Modular Questionnaire on Female Lower Urinary Tract Symptoms (ICIQ-FLUTS). The ICIQ group authorized the validation of the ICIQ-FLUTS questionnaire for Brazil.

### Participants

A total of 240 users of the urology and gynecology services in Maternidade Escola Januário Cicco of Federal University of Rio Grande do Norte (MEJC/UFRN) participated in the study. A subjects-to-variables ratio of 10:1 was considered as an adequate sample size. Therefore, a minimum of 240 women was required (12).

The inclusion criteria were: (a) be over 18 years of age, (b) with or without LUTS, and (c) not be pregnant or lactating. Those who: (a) gave up participating in the survey or withdrew their consent, or (b) presented an cognitive inability to respond to the questions were excluded from the research.

### Instruments

The ICIQ-FLUTS consists of 12 questions related to LUTS divided into three areas: filling (4 questions), voiding (3 questions), and incontinence (5 questions). The answers are based on experiences with LUTS in the previous four weeks. The score is calculated by domain and it ranges from zero to ten in each question (11).

Initially, all volunteers responded a form elaborated for this study with identification and sociodemographic data in addition to questions in relation to clinical aspects (use of medication, associated diseases, practice of physical activity, obstetrics and gynecology history).

The translation and adaptation of the ICIQ-FLUTS were carried out following standard guidelines (11).

1. Initial translation of the questionnaire into Portuguese was undertaken by two bilingual native speakers, independently. Synthesis of the translation by the two translators (V1).
2. Back translation into English by two native English speakers.
3. Review of the back translations by the ICIQ group and adjustment.
4. Spelling and semantic corrections for the creation of the second version in Portuguese (V2).

To verify the validity of the questionnaire (12), the V2 of the translated questionnaire was presented to a group of 5 patients who met the inclusion criteria of the study, as well as by a group of professionals which included physiotherapists, urologists and gynecologists. The observations made at this stage were performed, which resulted in small textual changes in the questions for better understanding.

Eighty (n=80) volunteers were randomly selected within the sample to participate in the inter-rater reliability analysis. The participants answered the ICIQ-FLUTS questionnaire for interviewer 1 and interviewer 2 after 30 minutes of finalizing the first evaluation. The order of the evaluators was always the same.

The stability of the questionnaire over time was verified with other group of eighty volunteers (n=80). The participants answered the questionnaire twice with an interval of 15 days between the evaluations. It is expected that LUTS will not undergo a significant change within its clinical condition during this period (12).

The validation was performed applying two questionnaires which had already been translated and validated in Brazil. The Utian Quality Of Life (UQOL) was validated for the Brazilian population by Lisboa et al. (13). Although the questions are not specific for the climacteric period, it is a questionnaire designed to quantify the quality of life during this phase of life. In total, there are 23 questions distributed in four domains on quality of life: occupational, health, sexual and emotional. The higher the score, the better the quality of life.

The other used questionnaire was the International Consultation on Incontinence Questionnaire - Short Form (ICIQ-SF). This questionnaire is a simple, brief and self-applied questionnaire, which assesses the impact of UI in quality of life and qualifies the urinary loss and it was translated and validated to Portuguese Language by Tamanini et al. (8). It consists of four questions that evaluate the frequency, severity and the impact of UI, in addition to a set of eight self-diagnosis items that allows to evaluate the causes or situations of UI experienced by the patients. The total score ranges from zero to twenty-one points and is divided into: no impact (0 point), light impact (1 to 3 points), moderate (4 to 6 points), severe (7 to 9 points) and very severe (10 or more points).

## Statistical analysis

Descriptive statistical was performed to present the information about sociodemographic and clinical aspects. The internal consistency assessment was performed using the Cronbach's alpha coefficient statistical test. The intra and inter-rater reliability were verified using the Intraclass Correlation Coefficient (ICC). The correlations were performed using the Pearson Correlation Test. The Statistical Package for Social Sciences version 20.0 program for Windows was used.

### Ethical issues

All the volunteers signed the Consent Term. The research was approved by the Ethics and Research Committee (number 1.846.197/2016) of the Federal University of Rio Grande do Norte.

## RESULTS

A total of 240 patients were interviewed, six of whom were excluded as they did not complete the questionnaire responses. 234 patients were included in the data analysis, with mean age of 50.96 years (±11.22) (CI: 49.28-52.65). The majority of the volunteers presented 1-2 Brazilian basic salary (45.4%), studied up to 10 years of schooling (30.5%) and had status marital married (59.7%).

The mean number of pregnancies was 3.18±0.15 (CI: 2.88-3.49), vaginal delivery was 2.09±0.14 (CI: 1.81 - 2.38) and cesarean section was 0.55±0.05 (CI: 0.44-0.67). According to hormonal status, 38.5% of the sample was in the reproductive phase, 8.3% in the menopausal transition period and 53.2% in the menopausal period. The time of menopause was 10.03±4.26 years. Table-1 shows the characteristics of the volunteers.

**Table 1:** Characteristics of the volunteers (n=234).

Variables		Number	Frequency (%)
**Educational level**			
	No study	49	21.5
	Up to 4 years	40	17.0
	5-8 years	24	10.5
	9-12 years	74	31.5
	Above 13 years	47	20.5
**Number of pregnancies**			
	Nulliparous	16	6.84
	1 pregnancy	101	43.16
	Above 1 pregnancy	117	50.00
**Types of delivery**			
	Vaginal	170	72.65
	Cesarean section	64	27.35
**Hormonal Status**			
	Reproductive phase	90	38.50
	Transition	19	8.12
	Menopausal period	125	53.38
**Menopause (n=125)**			
	Natural	78	62.50
	Surgical	47	37.50

The result of the Cronbach α coefficient of the instrument presented a value of 0.832. The inter-rater and intra-rater reliability are presented in Table-2.

**Table 2:** Intra and inter-rater reliability analysis of the ICIQ-FLUTS.

	Intra-rater reliability*	Inter-rater reliability*	Cronbach α coefficient
Question 2^a^	0.932	0.887	0.80
Question 2b	0.871	0.884	0.81
Question 3^a^	0.894	0.921	0.87
Question 2b	0.868	0.923	0.85
Question 4^a^	0.913	0.852	0.78
Question 4b	0.891	0.848	0.80
Question 5^a^	0.901	0.885	0.91
Question 5b	0.848	0.865	0.78
Question 6^a^	0.873	0.994	0.82
Question 6b	0.874	0.986	0.81
Question 7^a^	0.945	0.858	0.87
Question 7b	0.901	0.871	0.89
Question 8^a^	0.932	0.931	0.79
Question 8b	0.912	0.945	0.81
Question 9^a^	0.982	0.923	0.82
Question 9b	0.925	0.932	0.79
Question 10^a^	0.973	0.845	0.90
Question 10b	0.895	0.861	0.89
Question 11^a^	0.864	0.982	0.76
Question 11b	0.842	0.985	0.78
Question 12^a^	0.939	0.896	0.83
Question 12b	0.892	0.921	0.85
Question 13^a^	0.896	0.873	0.88
Question 13b	0.874	0.901	0.88

**ICIQ-FLUTS** = International Consultation on Incontinence Modular Questionnaire on Female Lower Urinary Tract Symptoms; **ICC** = Intraclass Correlation Coefficient

The correlation between ICIQ-FLUTS (score I - domain of urinary incontinence) with the ICIQ-SF (final score) was strong and positive (r=0.836, p=0.000). The ICIQ-FLUTS also showed moderate and negative correlation with the total score of UQOL (r=-0.691, p=0.017). The mean scores and correlation between the questionnaires are shown in Table-3.

**Table 3:** Mean final scores and correlation of the questionnaires ICIQ-FLUTS with ICIQ-SF and UQOL.

		Mean (SD)	CI	Correlation (r)
**ICIQ-FLUTS**				
	ScoreF	11.93 (±0.91)	10.12 - 13.74	-
	ScoreV	3.31 (±0.53)	2.25 - 4.36	-
	Score I	17.03 (±1.24)	14.57 - 19.49	-
	ICIQ-SF	9.13 (±0.57)	7.99 - 10.27	0.83
**UQOL**				
	Occupational	27.56 (±0.31)	19.62 - 20.59	-0.61
	Health	20.11 (±0.24)	19.44 - 22.44	-0.74
	Emotional	18.85 (±0.27)	18.30 - 19.40	-0.71
	Sexual	9.52 (±0.17)	9.17 - 9.87	-0.67
**Total**		**75.98 (±0.62)**	**74.75 - 77.22**	**-0.69**

**ICIQ-FLUTS** = International Consultation on Incontinence Modular Questionnaire on Female Lower Urinary Tract Symptoms; **UQOL** = Utian Quality of Life; ICIQ-SF = International Consultation on Incontinence Questionnaire - Short Form; **SD** = standard deviation; **CI** = confidence interval

## DISCUSSION

Throughout women's life, the onset of different types of LUTS is frequent though most evaluations or therapeutic approaches highlight only urinary incontinence among its symptoms. The ICIQ-FLUTS is a questionnaire which investigates female lower urinary tract symptoms by recording the degree of discomfort from the presence of this symptom. This questionnaire was chosen to be translated, adapted and validated for Portuguese because it is brief, applied over a few minutes, has a simple scoring method and identifies other urinary symptoms such as nocturia, dysuria and urgency. Moreover, it has already been translated for other languages such as Tamil (14), Greek (15), Chinese (16), Dutch, Danish, French, German, Korean, Norwegian and Swedish (11). The ICIQ-FLUTS validation process for the languages described above used ICIQ-SF and all results showed great reliability when related to the questionnaires.

The internal consistency of the ICIQ-FLUTS measured by the Cronbach α Coefficient was considered satisfactory. The ICIQ group (11) recommends 0.70 as the minimum acceptable value. In a recent validation study of the instrument for Tamil (14), this value was 0.80. Thus, we can demonstrate that the instrument has a good degree of correlation between its items.

The stability of the questionnaire was analyzed through the test-retest. The results demonstrate that the ICIQ-FLUTS has remained sensitive in measuring of LUTS over time. The interval of 15 days was chosen considering that the urinary symptoms should not undergo clinical changes (in the absence of treatment) in this time. Although, it is also expected that the participants do not remember the answers of the first evaluation. The values obtained in the Intraclass Correlation Coefficient test were good.

There was strong and positive correlation between ICIQ-FLUTS and ICIQ-SF. This result demonstrates that the ICIQ-FLUTS is reliably and satisfactorily evaluate UI. It is important to inform that ICIQ-SF is a questionnaire well-known for the scientific and clinical application of only urinary losses (17). Thus, ICIQ-FLUTS seems to be more complete in the wider investigation of other LUTS, since assesses effort and urgency UI, unlike other questionnaires that evaluate separately and focus only on UI.

The correlation between UQOL and ICIQ-FLUTS was moderate in the evaluation of the final score and domains. Perhaps some volunteers do not consider LUTS as a disease that affects their quality of life in various spheres of financial, social and personal aspects. In addition, some women believe that involuntary loss of urine is a natural aging process (18).

A limitation of this study was the need to read the questionnaire for a part of the sample (21.5%) that were illiterate. The users from the public health system in Brazil often have a low education level or are illiterate. Other study about validation of questionnaire corroborate this information (19, 20). The questionnaires by ICIQ group are self-administered.

More studies should be carried out to demonstrate the responsiveness of the questionnaire. It means to verify the sensitivity of the questionnaire before and after specific treatment for LUTS. However, the authors hope that the ICIQ-FLUTS can already be used in clinical and scientific research as part of screening for LUTS and monitoring its evolution.

## CONCLUSIONS

The Portuguese version of the ICIQ-FLUTS questionnaire showed strong correlation to ICIQ-SF questionnaire and satisfactory values to test––retest and internal consistency. Therefore, it is an important tool to detect LUTS among Brazilian female population.

## ABBREVIATIONS

LUTS: Lower Urinary Tract Symptoms
ICIQ-FLUTS: International Consultation on Incontinence Modular Questionnaire on Female Lower Urinary Tract Symptoms
UI: Urinary Incontinence
MEJC/UFRN: Maternidade Escola Januário Cicco of Federal University of Rio Grande do Norte
UQOL: Utian Quality Of Life
ICIQ-SF: International Consultation on Incontinence Questionnaire - Short Form
ICC: Intraclass Correlation Coefficient
CI: Confidence Interval

## References

[1] 1. Abrams P, Cardozo L, Fall M, Griffiths D, Rosier P, Ulmsten U, et al. The standardisation of terminology of lower urinary tract function: report from the Standardisation Sub-committee of the International Continence Society. Neurourol Urodyn. 2002;21:167-78. 10.1002/nau.10052 11857671

[2] 2. Coyne KS, Sexton CC, Thompson CL, Milsom I, Irwin D, Kopp ZS, et al. The prevalence of lower urinary tract symptoms (LUTS) in the USA, the UK and Sweden: results from the Epidemiology of LUTS (EpiLUTS) study. BJU Int. 2009;104:352-60. 10.1111/j.1464-410X.2009.08427.x 19281467

[3] 3. Irwin DE, Milsom I, Hunskaar S, Reilly K, Kopp Z, Herschorn S, et al. Population-based survey of urinary incontinence, overactive bladder, and other lower urinary tract symptoms in five countries: results of the EPIC study. Eur Urol. 2006;50:1306-14. 10.1016/j.eururo.2006.09.019 17049716

[4] 4. Soler R, Gomes CM, Averbeck MA, Koyama M. The prevalence of lower urinary tract symptoms (LUTS) in Brazil: Results from the epidemiology of LUTS (Brazil LUTS) study. Neurourol Urodyn. 2018;37:1356-64. 10.1002/nau.23446 29106747

[5] 5. Bilgic D, Beji NK. Lower urinary tract symptoms in women and quality of life. International Journal of Urological Nursing. 2010;4:97-105.

[6] 6. Jackson S, Donovan J, Brookes S, Eckford S, Swithinbank L, Abrams P. The Bristol Female Lower Urinary Tract Symptoms questionnaire: development and psychometric testing. Br J Urol. 1996;77:805-12. 10.1046/j.1464-410x.1996.00186.x 8705212

[7] 7. Brookes ST, Donovan JL, Wright M, Jackson S, Abrams P. A scored form of the Bristol Female Lower Urinary Tract Symptoms questionnaire: data from a randomized controlled trial of surgery for women with stress incontinence. Am J Obstet Gynecol. 2004;191:73-82. 10.1016/j.ajog.2003.12.027 15295345

[8] 8. Tamanini JT, Dambros M, D'Ancona CA, Palma PC, Rodrigues Netto N Jr. Validation of the “International Consultation on Incontinence Questionnaire - Short Form” (ICIQ-SF) for Portuguese. Rev Saude Publica. 2004;38:438-44. 10.1590/s0034-89102004000300015 15243675

[9] 9. Tamanini JT, D'Ancona CA, Botega NJ, Rodrigues Netto N Jr. Validation of the Portuguese version of the King's Health Questionnaire for urinary incontinent women. Rev Saude Publica. 2003;37:203-11. 10.1590/s0034-89102003000200007 12700842

[10] 10. Pereira VS, Santos JY, Correia GN, Driusso P. [Translation and validation into Portuguese of a questionnaire to evaluate the severity of urinary incontinence]. Rev Bras Ginecol Obstet. 2011;33:182-7. 10.1590/s0100-72032011000400006 21845350

[11] 11. Bristol Urological Institute, Internacional Consultation on Incontinence Modular Questionnaire (ICIQ). Available at. <http://iciq.net>. Acessed 15 March 2018.

[12] 12. Bryant FB, Yarnold PR. Principal-components analysis and exploratory and confirmatory factor analysis. In: Grimm LG, Yarnold PR, editors. Reading and understanding multivariate statistics. Washington DC: American Psychological Association. 1995:99-136.

[13] 13. Lisboa LL, Utian W, da Fonseca Filho GG, de Azevedo GD. Translation, adaptation and validation of the Brazilian version of the Utian Quality of Life for evaluation of quality of life in the climacteric. Rev Bras Ginecol Obstet. 2015;37:520-5. 10.1590/SO100-720320150005438 26561242

[14] 14. Ekanayake CD, Pathmeswaran A, Nishad AAN, Samaranayake KU, Wijesinghe PS. Translation and validation of ICIQ-FLUTS for Tamil-speaking women. Int Urogynecol J. 2017;28:1875-81. 10.1007/s00192-017-3316-5 28343316

[15] 15. Athanasiou S, Grigoriadis T, Kyriakidou N, Giannoulis G, Antsaklis A. The validation of international consultation on incontinence questionnaires in the Greek language. Neurourol Urodyn. 2012;31:1141-4. Erratum in: Neurourol Urodyn. 2012;31:1310. 10.1002/nau.22197 22508384

[16] 16. Huang L, Zhang SW, Wu SL, Ma L, Deng XH. The Chinese version of ICIQ: a useful tool in clinical practice and research on urinary incontinence. Neurourol Urodyn. 2008;27:522-4. 10.1002/nau.20546 18351586

[17] 17. Abrams P, Andersson KE, Birder L, Brubaker L, Cardozo L, Chapple C, et al. Fourth International Consultation on Incontinence Recommendations of the International Scientific Committee: Evaluation and treatment of urinary incontinence, pelvic organ prolapse, and fecal incontinence. Neurourol Urodyn. 2010;29:213-40. 10.1002/nau.20870 20025020

[18] 18. Shah D, Badlani G. Treatment of overactive bladder and incontinence in the elderly. Rev Urol. 2002;4 Suppl 4:S38-43. PMC1476020 16986020

[19] 19. Moraes RP, Silva JLD, Calado AA, Cavalcanti GA. Validation of the urgency questionnaire in Portuguese: A new instrument to assess overactive bladder syndrome. Int Braz J Urol. 2018;44:338-347. 10.1590/S1677-5538.IBJU.2017.0147 PMC6050559 29219282

[20] 20. Pereira SB, Thiel Rdo R, Riccetto C, Silva JM, Pereira LC, Herrmann V, et al. Validation of the International Consultation on Incontinence Questionnaire Overactive Bladder (ICIQ-OAB) for Portuguese. Rev Bras Ginecol Obstet. 2010;32:273-8. 10.1590/s0100-72032010000600004 20945012

